# Prevalence and management of driveline infections in mechanical circulatory support - a single center analysis

**DOI:** 10.1186/s13019-021-01589-6

**Published:** 2021-08-03

**Authors:** Andrzej Juraszek, Mikołaj Smólski, Piotr Kołsut, Jarosław Szymański, Paweł Litwiński, Krzysztof Kuśmierski, Joanna Zakrzewska-Koperska, Maciej Sterliński, Tomasz Dziodzio, Mariusz Kuśmierczyk

**Affiliations:** 1 Department of Cardiac Surgery and Transplantation, The Cardinal Stefan Wyszyński National Institute of Cardiology, ul. Alpejska 42, 04-628 Warsaw, Poland; 2 Medical University of Warsaw, Warsaw, Poland; 3 1st Department of Arrhythmia, The Cardinal Stefan Wyszyński National Institute of Cardiology, Warsaw, Poland; 4 Department of Surgery, Campus Charité Mitte and Campus Virchow-Klinikum Charité –Universitätsmedizin Berlin, corporate member of Freie Universität Berlin, Humboldt-Universität zu Berlin, and Berlin Institute of Health, Augustenburger Platz 1, 13353 Berlin, Germany

**Keywords:** Driveline infection, Left ventricular assist device, Surgical reposition

## Abstract

**Background:**

Driveline infections in continuous-flow left ventricular assist devices (cf-LVAD) remain the most common adverse event. This single-center retrospective study investigated the risk factors, prevalence and management of driveline infections.

**Methods:**

Patients treated after cf-LVAD implantation from December 2014 to January 2020 were enrolled. Baseline data were collected and potential risk factors were elaborated. The multi-modal treatment was based on antibiotic therapy, daily wound care, surgical driveline reposition, and heart transplantation. Time of infection development, freedom of reinfection, freedom of heart transplantation, and death in the follow-up time were investigated.

**Results:**

Of 75 observed patients, 26 (34.7%) developed a driveline infection. The mean time from implantation to infection diagnosis was 463 (±399; range, 35–1400) days. The most common pathogen was *Staphylococcus aureus* (*n* = 15, 60%). First-line therapy was based on antibiotics, with a primary success rate of 27%. The majority of patients (*n* = 19; 73.1%) were treated with surgical reposition after initial antibiotic therapy. During the follow-up time of 569 (±506; range 32–2093) days, the reinfection freedom after surgical transposition was 57.9%. Heart transplantation was performed in eight patients due to resistant infection. The overall mortality for driveline infection was 11.5%.

**Conclusions:**

Driveline infections are frequent in patients with implanted cf-LVAD, and treatment does not efficiently avoid reinfection, leading to moderate mortality rates. Only about a quarter of the infected patients were cured with antibiotics alone. Surgical driveline reposition is a reasonable treatment option and does not preclude subsequent heart transplantation due to limited reinfection freedom.

**Supplementary Information:**

The online version contains supplementary material available at 10.1186/s13019-021-01589-6.

## Background

The success of continuous-flow left ventricular assist device (cf-LVAD) therapy is reduced by several severe complications like hemorrhagic and thromboembolic events, arrhythmias, multi-organ failure, and driveline infections [1–4]. Driveline infections are the most common adverse event, occurring in about 28% of all patients with an implanted cf-LVAD [4, 5]. It is suspected that the transcutaneous pathway of the driveline and the chronic traumatic conditions are the triggers for infections [3]. A negative impact on the necessity of reoperation, ascending strokes, postponing transplantation, and survival is described in literature [3, 6]. Conservative management is based on the use of antibiotic therapy, and the surgical approach includes the use of vacuum dressings and driveline reposition. If all other strategies fail, heart transplantation is applied as last treatment resort [6, 7]. Currently, data regarding the treatment strategies and outcome of complications related to driveline infections is limited [6–12].

### Aim of the study

This retrospective single-site study investigated the risk factors of driveline infections and the results of their medical and surgical treatments in cf-LVAD patients.

## Methods

### Patients and data

All patients treated after cf-LVADs implantation from December 2014 to January 2020 in our center were enrolled. The study was approved by ethics committee. Patients provided written informed consent to participate in the study. The following cf-LVADs were implanted as bridge to transplantation therapy: HeartWare (Medtronic, Minneapolis, MN, USA), Heartmate II and HeartMate III (both Abbott, Abbott Park, IL, USA). Baseline data included patients’ age and gender, implantation indication, and device type. Specific data included time from cf-LVAD implantation to driveline infection, pathogen type, antibiotic treatment strategy, and time from diagnosis to surgical reposition. The freedom of reinfection in the follow-up time was investigated.

Risk factors for the development of driveline infections were assessed individually, including: obesity, defined as body mass index (BMI) > 30 kg/m^2^; diabetes mellitus; age < 45 years; intensive care unit (ICU) stay over 2 weeks; history of previous mechanical circulatory support (MCS), defined as any type of left ventricle support implanted before the infection-associated cf-LVAD device, including short-term left ventricle support; chronic kidney disease, defined as abnormally elevated serum creatinine for more than 3 months or calculated glomerular filtration rate (GFR) < 60 mL/min/1.73m^2^. Finally, the number of heart transplantations due to driveline infections and overall mortality were recorded.

### Regular driveline exit care in non-infected patients

Changing the dressing every 2–3 days and always after exposure to water was recommended. In case of efflux on the driveline exit site, daily dressing was necessary.

For the dressing change procedure disposable sterile gloves were used. Three separate sterile packets of gauze pads were placed on a clean surface. One part was soaked in a disinfectant that can be used on open wounds (Octanisept®, Schülke & Mayr GmbH, Norderstedt, Germany), the second part was soaked in a disinfectant liquid containing the active substances: 2-propanol, 1-propanol, 2-diphenylol (Kodan®, Schülke & Mayr GmbH, Norderstedt, Germany), leaving the third part dry. The soaked Kodan gauze was put on the driveline exit point and left there for at least two minutes. After the specified time, the gauze pad was removed from the driveline exit point and the driveline exit place was cleaned with Octanisept®, using movements in direction from the wound outwards. The area around the wound was dried while avoiding the cable exit area. As a standard practice, we used an antiseptic and antimicrobial dressing impregnated with polyhexamethylene biguanide. It was a dressing with an additional cutout to cover the cable exit point. Finally, covering of the central part with a transparent and breathable foil dressing was required, to separate the wound from the environment. In order to protect the driveline against enhanced movement, additional fixing elements were added.

### Treatment strategy

The treatment strategy comprised of three stages: antibiotic therapy, surgical driveline reposition, and heart transplantation. Antibiotics were used as first-line therapy. The first choice antibiotic agent was empiric and then adjusted to the culture results. Additionally, daily wound care comprising of local antiseptic application and dressing replacement was performed in each stage.

**Fig. 1:**
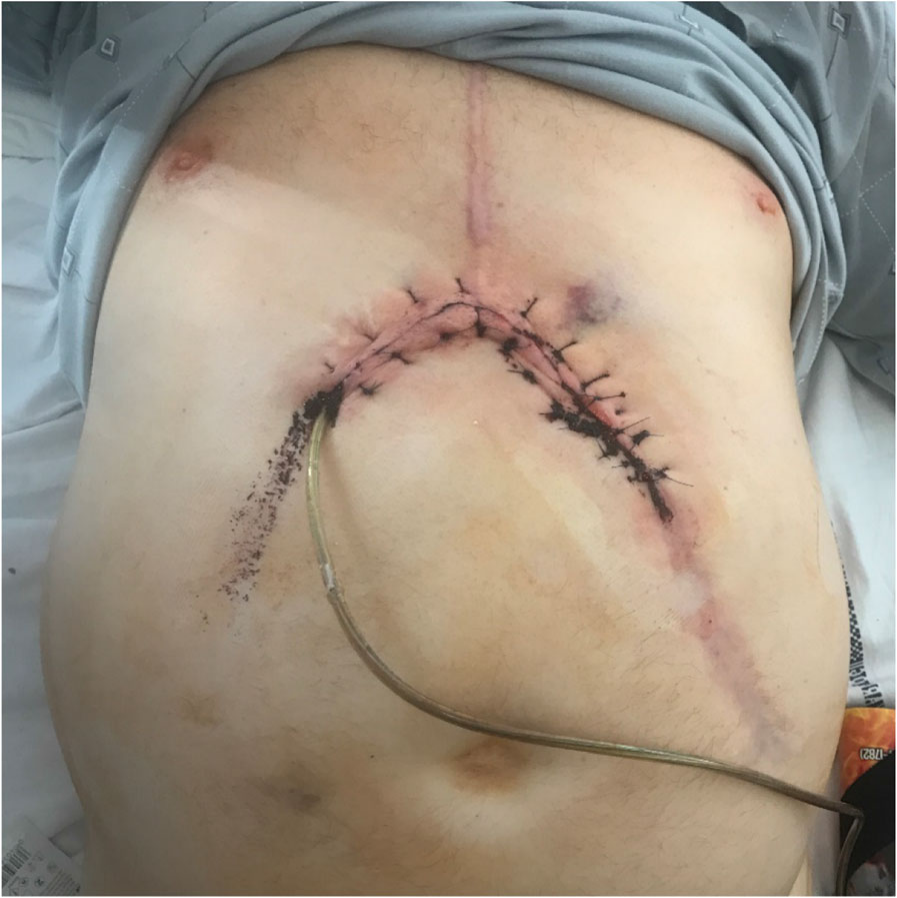
The postoperative view after driveline reposition

The surgical approach was chosen in resistant infections. The surgery was performed under general anesthesia. The skin was cut above the run of the driveline, and the velvet cover was removed from the driveline. Depending on the proximity of infection, the former driveline site was treated with a vacuum dressing or sutured secondarily. Primary wound closure was performed in proximal infections. In the case of vacuum therapy, the wound was sutured secondly. Similarly, depending on the degree of the infection, a new incision was made on the contralateral side of the abdomen, creating a new driveline site either in the first or second surgery. The skin over the new driveline placement was then sutured (Fig. 1). Prolonged suppressive antibiotic therapy was used.

In cases of very resistant infections, patients were placed on a list for urgent heart transplantation as last treatment resort.

### Statistical methods

Driveline infection risk factors were described using odds ratios (OR) with respective 95% confidence intervals (CI). Because of sample size we decided to perform only univariate analyses. Time of infection freedom and time to transplantation or death were described using Kaplan–Meier estimates. Log-rank test was performed to compare patient groups. All analyses were conducted using R 4.0.2 statistical software (R Core Team (2020). R: A language and environment for statistical computing. R Foundation for Statistical Computing, Vienna, Austria. https://www.R-project.org/).

## Results

### Patients and data

**Fig. 2:**
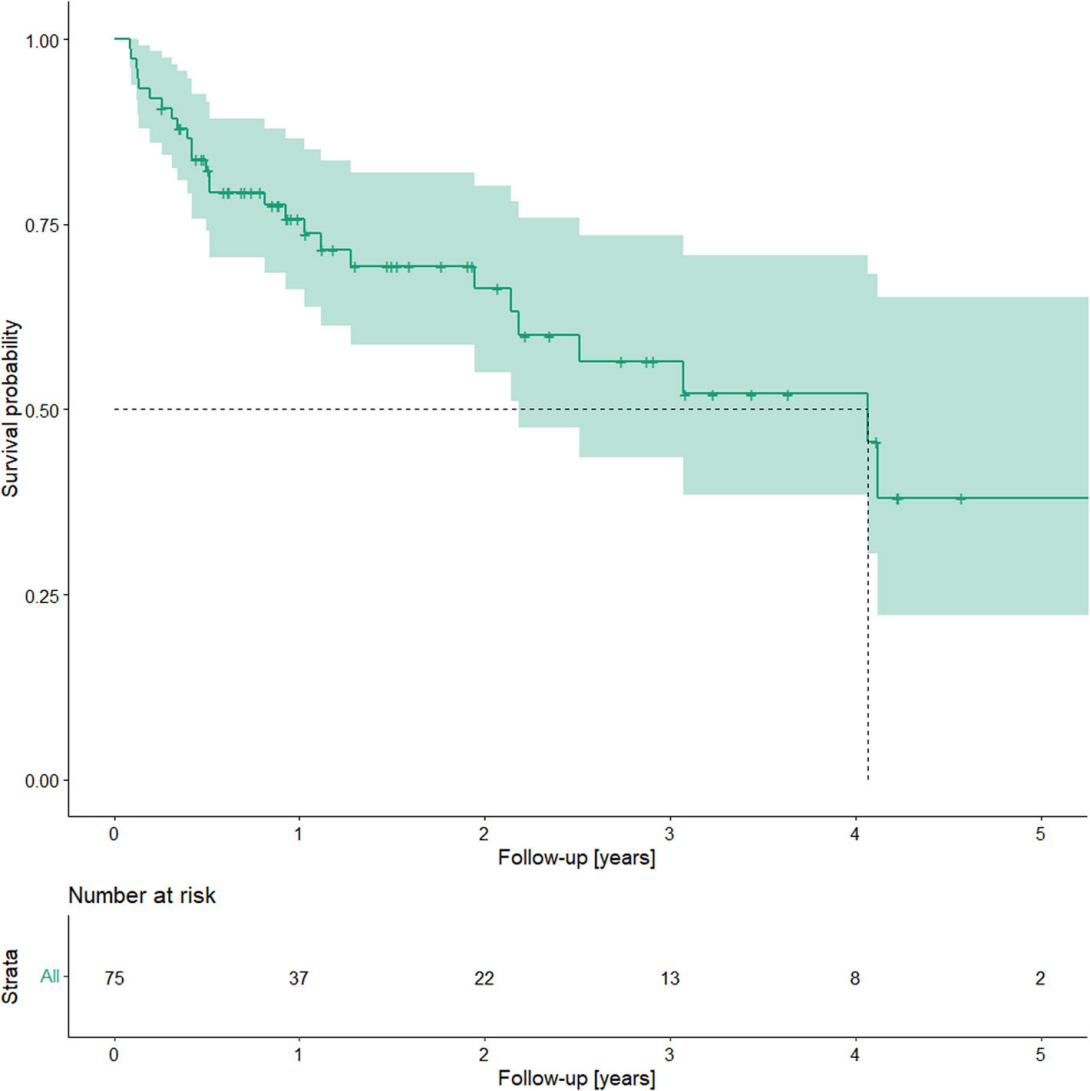
A Kaplan–Meier plot of infection freedom after cf-LVAD

During the study period, 75 patients including 71 males; mean age, 54 (±12.9; range 12–68.6) years, were treated after cf-LVAD implantation (HeartWare, *n* = 34, 45.3%; HeartMate II, *n* = 5, 6.7%; HeartMate III, *n* = 36, 48%). Twenty-six (34.7%) developed a driveline infection (HeartWare, *n* = 13, 50%; HeartMate III, *n* = 11, 42.3%; HeartMate II, *n* = 2, 7.7%). The median follow-up time was 2.13 years. The mean time from implantation to infection diagnosis was 463(±399) days (range, 35–1400 days). A Kaplan–Meier plot of infection freedom is shown in Fig. 2.

The potential risk factors for driveline infection were analyzed in Table 1. An ICU stay longer than 2 weeks was the only significant protective factor against driveline infection. The most common pathogen was *Staphylococcus aureus* (*n* = 15, 60%). Cloxacillinum was the most frequently used antibiotic (*n* = 10, 31.3%). The majority of patients received more than one antibacterial agent. The particular pathogens and antibiotics used in each patient are listed in Table 2.

**Table 1:** Risk factors for driveline infection

Risk factors present at driveline infection diagnosis	Number (%) of patients with or without driveline infection		OR (95% CI, p)
	Infection	No Infection	
Obesity	8 (25.8)	23 (74.2)	0.50 (0.18–1.34, *p* = 0.179)
Diabetes mellitus	12 (35.3)	22 (64.7)	1.05 (0.40–2.74, *p* = 0.917)
Age < 45 years	6 (46.2)	7 (53.8)	1.80 (0.52–6.12, *p* = 0.342)
ICU stay > 2 weeks	7 (20.6)	27 (79.4)	0.30 (0.10–0.82, *p* = 0.022)
History of previous mechanical circulatory support	2 (18.2)	9 (81.8)	0.37 (0.05–1.59, *p* = 0.228)
Chronic kidney disease	9 (28.1)	23 (71.9)	0.60 (0.22–1.58, *p* = 0.306)

*CI* confidence interval, *OR* univariate odds ratio

**Table 2:** Detected pathogens and antibiotics used in particular patients

Patient	Pathogen	Antibiotic 1	Antibiotic 2	Antibiotic 3	Antibiotic 4	Antibiotic 5
**1**	MSSA	Clindamycinum	Cloxacillinum	–	–	–
**2**	MSSA	Cloxacillinum	–	–	–	–
**3**	MSSA	Ciprofloxacinum	Cefuroxime	–	–	–
**4**	MSSA	Cloxacillinum	–	–	–	–
**5**	*P. mirabilis*	Ceftriaxone	–	–	–	–
**6**	*S. agalactiae*	Cefadrioxil	–	–	–	–
**7**	MSSA	Cloxacillinum	Cefuroxime	–	–	–
**8**	*K. pneumoniae*	Piperacillin + Tazobactam	Cefepime	–	–	–
**9**	MSSA	Vancomycin	Clindamycin	–	–	–
**10**	MRSA	Linezolid	–	–	–	–
**11**	MSSA	Ceftazidime	Cloxacillinum	–	–	–
**12**	*S. epidermidis*	Ciprofloxacinum	Vancomycin	Cloxacillinum	Rifampicin	Imipenem +Cilastatin
**13**	K. pneumoniae	Cefepime	–	–	–	–
**14**	UNKNOWN	UNKNOWN	–	–	–	–
**15**	MRSA	Vancomycin	–	–	–	–
**16**	UNKNOWN	UNKNOWN	–	–	–	–
**17**	MSSA	Cloxacillinum	–	–	–	–
**18**	MSSA	Cloxacillinum	Vancomycin	–	–	–
**19**	*S. pyogenes*	Cefuroxime	–	–	–	–
**20**	MSSA	Ciprofloxacinum	–	–	–	–
**21**	MSSA	Cloxacillinum	–	–	–	–
**22**	*P. aeruginosa*	Meropenem	Linezolid	Ampicillin	–	–
**23**	MSSA	Vancomycin	Meropenem	Cloxacillinum	–	–
**24**	P. aeruginosa	Piperacillin + Tazobactam	–	–	–	–
**25**	MRSA/P. aeruginosa	Linezolid	Ceftazidime	–	–	–
**26**	P. aeruginosa	Meropenem	–	–	–	–

### Treatment strategy

**Fig. 3:**
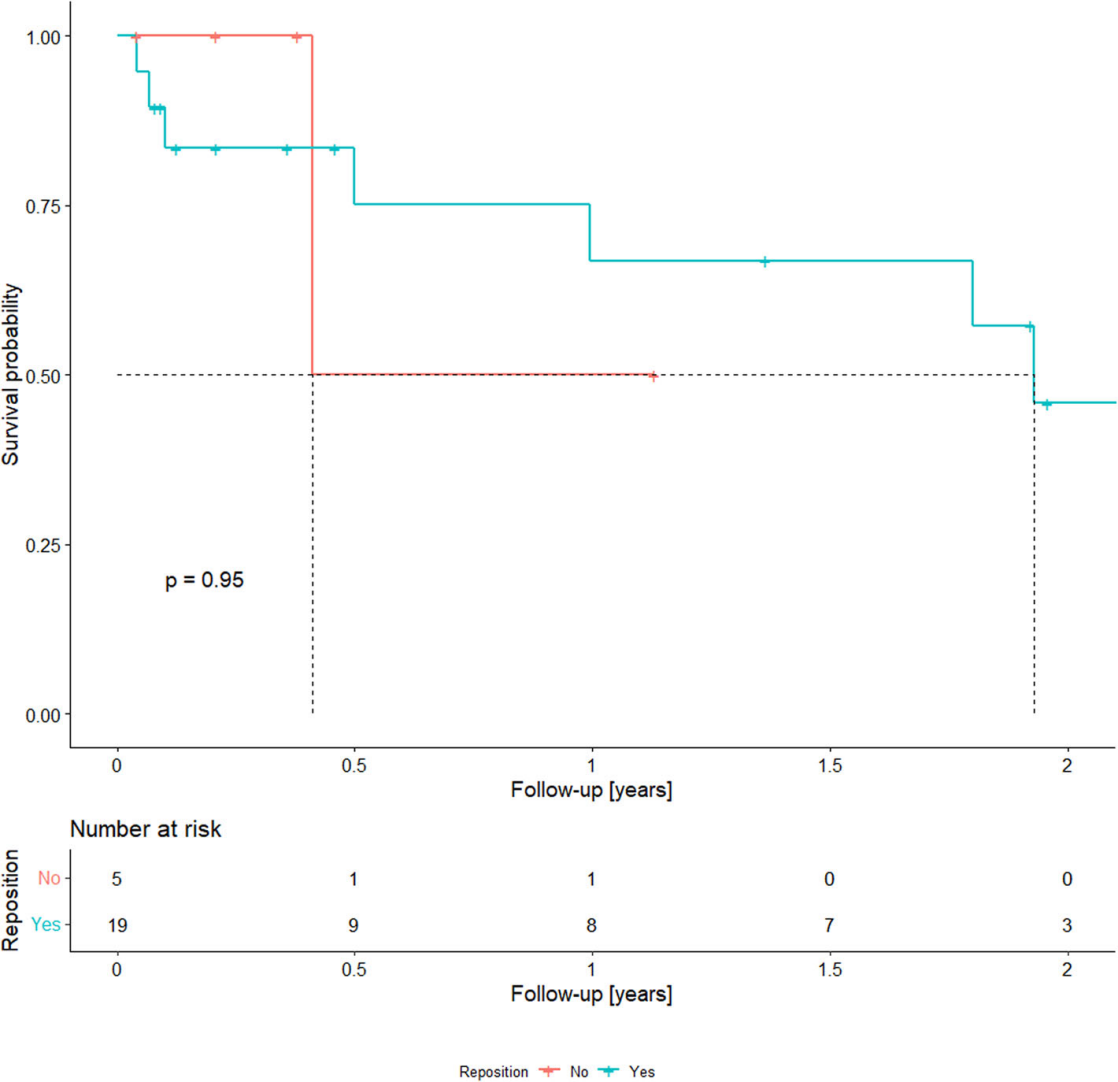
A Kaplan–Meier plot of reinfection freedom after driveline infection depending on treatment type

All patients were initially treated with antibiotics. The majority (19 patients, 73.1%) were finally treated with surgical reposition (reposition group), and seven patients (26.9%) were treated with antibiotics only (antibiotics group). In the reposition group, the mean time from diagnosis to surgical revision was 87(±136) days (range, 6–555 days). Thirteen patients in the reposition group were additionally treated with vacuum dressing. Follow-up time after infection treatment was 569(±506) days (range, 32–2093 days). Among seven patients with infections managed with antibiotics alone, one patient (14.3%) developed a reinfection. Of the 19 patients treated with surgical reposition, eight (42.1%) developed a reinfection. Reinfection rates in the antibiotic and reposition groups are shown in Table 3. The reinfection freedom rate is shown in Fig. 3.

**Table 3:** Reinfection rates in the antibiotic and surgical reposition groups

		n	Reinfection			
			No		Yes	
**Reposition**	**No**	7	6	85.7%	1	14.3%
	**Yes**	19	11	57.9%	8	42.1%

*P* = 0.36 (Fischer’s test)

**Fig. 4:**
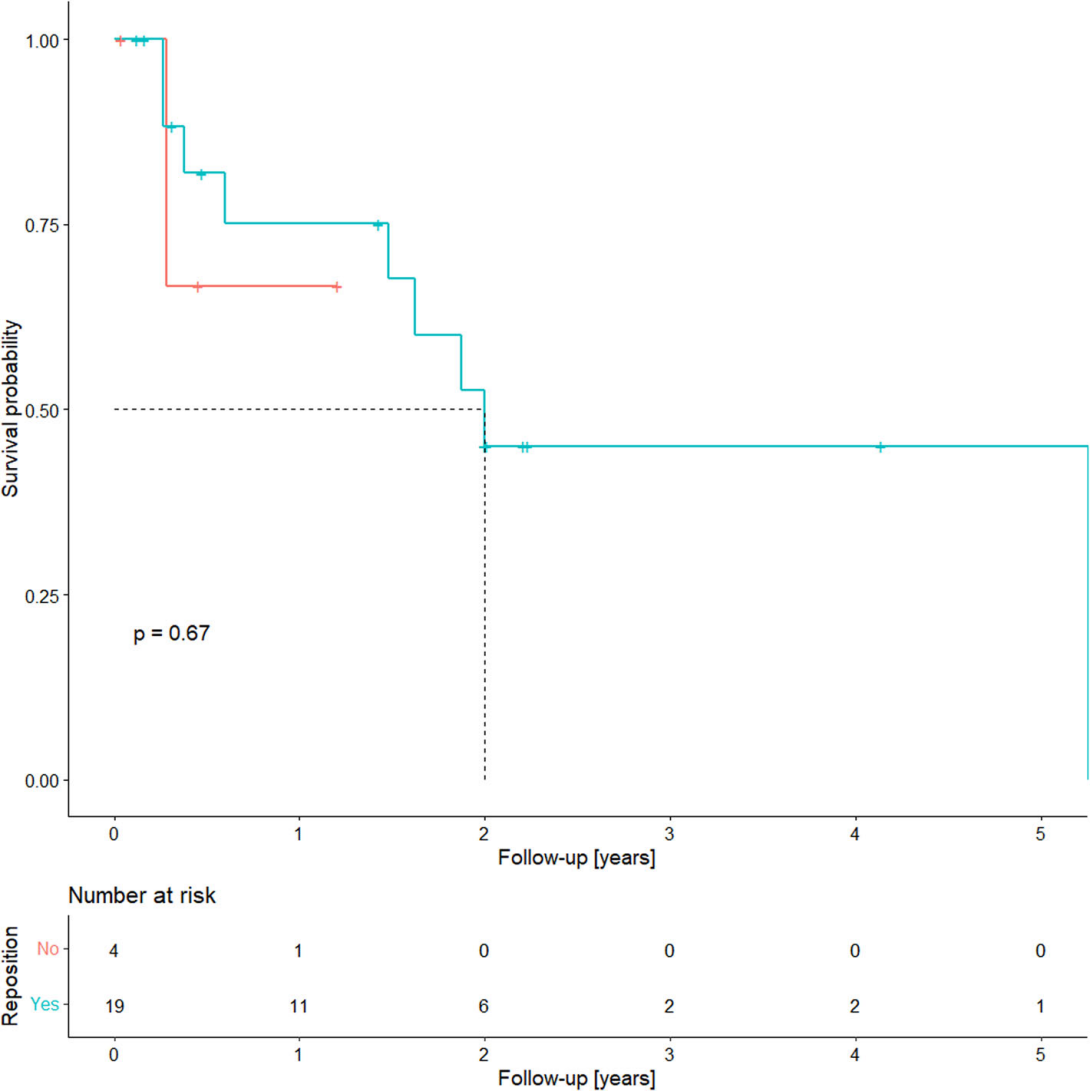
A Kaplan–Meier plot of freedom from transplantation or death after driveline infection depending on treatment type

One death due to recurrent driveline infection occurred (Fig. 4). Eight patients (30.7%) were treated with heart transplantation. Thirty-day mortality after urgent heart transplantation was 25%. The overall mortality for driveline infection was 11.5%.

## Discussion

We investigated the risk factors and results of medical and surgical treatment for driveline infections in cf-LVAD patients. Driveline infection was highly prevalent in cf-LVAD patients. We used a multi-modal strategy for driveline infection treatment. The first-line therapy was based on antibiotics, with a 27% primary success rate. The second step was surgical reposition, with a 57.9% primary success rate. Finally, heart transplantation was performed in 30.8% of patients for resistant infections. The infection-related mortality rate was moderate (11.5%). This is a retrospective observational study; therefore, routines and decisions cannot be compared and may vary between subjects.

In our series, the incidence of driveline infections was 34.7%, which is similar to other reports [8]. In our center, we use accurate, daily monitoring of patients using our own telemonitoring application and regular dressing changing. This may result in a prolonged time from implantation to the development of infection, which was longer than a year in our group. In comparison, the median time from cf-LVAD implantation to the first infection event was 291 days in the MOMENTUM trial cohort [4].

Although preventing driveline infection is important, an international standard for the prevention of driveline infection after the perioperative period has not yet been defined, and many centers apply their own protocol for driveline exit site care. However, in March 2019, cf-LVAD coordinators and cardiac surgeons from Germany and Austria prepared a 10-step procedure for driveline care. An advanced wound staging approach was defined with recommended actions for the prevention, early detection, and stage-related management of driveline infections [12].

Another proposed preventive double tunnel driveline technique includes placement of the driveline in the sheath of the rectus muscle in the umbilical direction and then subcutaneously to the left upper quadrant. This technique leads to significantly lowered infection rates. Indeed, 5 years after cf-LVAD implantation, the infection rate of patients operated by a double tunneling technique was 30%, compared to 61% after the conventional technique [13].

As the most common complication, driveline infection remains one of the most limiting factors in cf-LVAD therapy. Recurrent and prolonged hospitalizations significantly affect the patients’ quality of life. Several risk factors for the infective process have been described, most commonly, obesity [14] and younger age [15]. Particularly, younger age is associated with higher physical activity, which leads to more driveline irritation and tension. Another described issue is the velour coating on the exit site of the driveline [16]. For this reason, we performed an excessive debridement of the velour coating during the reposition surgery. This process should be performed very carefully to avoid intraoperative driveline damage. Moreover, a prolonged stay in the ICU cannot be considered a protective factor against infection in clinical practice, although we found it was statistically significantly associated with reduced infection in our analysis. The only explanation for this relationship may be the lower mobility of patients in the intensive care unit. This condition can reduce the irritation of the driveline. However, extending the stay in the intensive care unit may not be determined by the desire to avoid driveline infection. Interestingly, obesity was not recognized as a risk factor for developing driveline infection. This confirms the significant role of irritation and increased mobility in the development of driveline infections.

The low number of patients cured with antibiotics alone in our center indicates the ineffectiveness of this method. Moreover, although an aggressive surgical strategy of driveline debridement and reposition of the driveline exit site is a reasonable treatment option, the results of surgical reposition are limited. Our preliminary results of 45 patients in the observation period from 2014 to 2019 suggested surgical reposition were promising, with only 20% of patients treated surgically who developed reinfection during the follow-up time of 425(±487) days, range, 38–1644 days. However, after a follow-up of more than 2 years, the reinfection rate has increased to almost 60% [17].

Being a growing cf-LVAD center, we were forced to perform heart transplantation in very resistant infections. Urgent transplantation was characterized by significant early mortality. We have not yet used the relocation technique with the omentum. Although it seems to be a feasible and effective procedure in selected cases, the reported risk for perioperative bleeding is significant [18]. Interestingly, in the study performed by Radcliffe et al., chronic, prolonged antibiotic therapy (mean 486 days; range, 48–2287 days) led to successful outcomes in 50% of patients [11]. Therefore, significantly prolonged antibiotic therapy may be required in cf-LVAD patients.

Although the surgical driveline reposition is a viable treatment option, our strategy has moved towards antibiotics administration over an extended period of time as a first-line treatment. We had hoped that the surgical reposition technique was so effective that it would avoid heart transplantation due to infection; however, we were still forced to perform heart transplantation in a case of resistant infection as the last treatment resort. The management of a particular cf-LVAD center should be subject to continuous analysis of the results to optimize the treatment strategy.

Finally, competitively to heart transplantation with a limited donor pool, some innovative strategies should be considered. For example, cold atmospheric plasma application is a new tool for the treatment of superficial driveline infections. Cold atmospheric plasma creates reactive oxygen and nitrogen species that can inactivate microorganisms, including multi-resistant strains. Indeed, Hilker et al. reported a case of a patient with HeartWare cf-LVAD, in which the local infection was completely healed after 12 applications of cold plasma [19]. However, this promising approach requires further confirmation in a larger cohort of patients.

## Conclusions

Although the prevalence of driveline infection was high in our center, and treatment did not efficiently avoid reinfection, it was associated with moderate mortality. Only a minority of infected patients were cured with antibiotics alone, and the results of surgical driveline reposition were limited. For those reasons, heart transplantation is becoming the ultimate therapy in a growing number of patients. Nonetheless, the management of a particular cf-LVAD center should be subject to continuous analysis of the results to optimize the multi-modal treatment strategy. In the future, competitively to heart transplantation with a limited donor pool, alternative approaches should be developed.

### Limitations

Retrospective nature of the study, limitation to a single care center.

## Supplementary Information

**Additional file 1.** Pathogens and detailed antibiotic therapy.

## Data Availability

The datasets used and/or analysed during the current study are available from the corresponding author on reasonable request.

## Abbreviations

cf-LVAD: Continuous-flow left ventricular assist devices
BMI: Body mass index
ICU: Intensive care unit
MCS: Mechanical circulatory support
GFR: Glomerular filtration rate
