# Supplementary material for: Prevalence and management of driveline infections in mechanical circulatory support - a single center analysis
Source: J Cardiothorac Surg. 2021 Aug 3;16:216. doi: 10.1186/s13019-021-01589-6 (PMC8335934; doi:10.1186/s13019-021-01589-6)
Supplement: Supplementary file 1 — Additional file 1. Pathogens and detailed antibiotic therapy. [file 13019_2021_1589_MOESM1_ESM.docx]

**Supplementary data**

Pathogens and detailed antibiotic therapy.

| **Patient** | **Pathogen** | **Antibiotic 1** | **Antibiotic 2** | **Antibiotic 3** | **Antibiotic 4** | **Antibiotic 5** |
| --- | --- | --- | --- | --- | --- | --- |
| **1** | MSSA | Clindamycin | Cloxacillinum I.V. 1G q.i.d. for 20 days followed by Cloxacillinum 0.5G p.o. q.i.d. for 9 days | - | - | - |
| **2** | MSSA | Cloxacillinum 2G I.V. q.4h for 25 days | - | - | - | - |
| **3** | MSSA | Ciprofloxacinum | Cefuroxime | - | - | - |
| **4** | MSSA | Cloxacillinum 1G I.V. q.4h for 13 days | - | - | - | - |
| **5** | P. mirabilis | Ceftriaxone 1G I.V. b.i.d. for 18 days | - | - | - | - |
| **6** | S. agalactiae | Cefadroxil 0,5G p.o. b.i.d. for 8 days | - | - | - | - |
| **7** | MSSA | Cloxacillinum 1G I.V. q.4h for 27 days | Cefuroxime | - | - | - |
| **8** | K. pneumoniae | Piperacillin + Tazobactam 4G + 0,5G I.V. t.i.d. for 8 days | Cefepime 1G I.V. t.i.d. for 9 days | - | - | - |
| **9** | MSSA | Vancomycin-MIP 1G I.V. b.i.d. for 8 days | Clindamycin 0,3G p.o. t.i.d. for 8 days | - | - | - |
| **10** | MRSA | Linezolid | - | - | - | - |
| **11** | MSSA | Ceftazidime 1G I.V t.i.d. for 8 days | Cloxacillinum 1G I.V. q.i.d. for 26 days | - | - | - |
| **12** | S. epidermidis | Ciprofloxacinum 500MG p.o. t.i.d. for 9 days | Vancomycin-MIP 1G I.V. b.i.d. for 4 days | Cloxacillinum 2G I.V. q.4h for 17 days | Rifampicin 600MG p.o. b.i.d. for 15 days | Imip­enem +Cilastatin 20ML I.V. t.i.d. for 27 days |
| **13** | K. pneumoniae | Cefepime | - | - | - | - |
| **14** | UNKNOWN | UNKNOWN | - | - | - | - |
| **15** | MRSA | Vancomycin-MIP 500 I.V. t.i.d. for 3 days followed by b.i.d. for 4 days | - | - | - | - |
| **16** | UNKNOWN | UNKNOWN | - | - | - | - |
| **17** | MSSA | Cloxacillinum 1G I.V. q.4h for 5 days | - | - | - | - |
| **18** | MSSA | Cloxacillinum | Vancomycin | - | - | - |
| **19** | S. pyogenes | Cefuroxime | - | - | - | - |
| **20** | MSSA | Ciprofloxacinum 500MG p.o. b.i.d. for 14 days | - | - | - | - |
| **21** | MSSA | Cloxacillinum 2G I.V. q.4h for 30 days | - | - | - | - |
| **22** | P. aeruginosa | Meropenem | Linezolid | Ampicillin | - | - |
| **23** | MSSA | Vancomycin-MIP 500 I.V. q.i.d. for 5 days | Meropenem | Cloxacillinum 2G I.V. q.4h for 8 days | - | - |
| **24** | P. aeruginosa | Piperacillin + Tazobactam 4G + 0,5G I.V. t.i.d. for 31 days | - | - | - | - |
| **25** | MRSA/P. aeruginosa | Linezolid 0.6G/300ML I.V. b.i.d. for 6 days | Ceftazidime 2G I.V. b.i.d. for 6 days | - | - | - |
| **26** | P. aeruginosa | Meropenem | - | - | - | - |

Abbreviations:
I.V. – intravenous
p.o. - orally
b.i.d. – twice a day
t.i.d. – three times a day
q.i.d. – four times a day
q.4h – every 4 hours
